# m^6^A Methyltransferase KIAA1429 Regulates the Cisplatin Sensitivity of Gastric Cancer Cells via Stabilizing FOXM1 mRNA

**DOI:** 10.3390/cancers14205025

**Published:** 2022-10-14

**Authors:** Zhongcheng Zhu, Yuan Zhou, Yongheng Chen, Zhongyi Zhou, Wenxue Liu, Linyi Zheng, Qian Pei, Fengbo Tan, Haiping Pei, Yuqiang Li

**Affiliations:** 1 Department of General Surgery, Xiangya Hospital, Central South University, 87 Xiangya Road, Changsha 410008, China; 2 National Clinical Research Center for Geriatric Disorders, Xiangya Hospital, Central South University, 87 Xiangya Road, Changsha 410008, China; 3 NHC Key Laboratory of Cancer Proteomics, Laboratory of Structural Biology, Xiangya Hospital, Central South University, Changsha 410008, China; 4 Department of Geriatric Medicine, Xiangya Hospital, Central South University, Changsha 410008, China

**Keywords:** m^6^A modification, drug resistance, mRNA stability, p65, YTHDF1

## Abstract

**Simple Summary:**

N6-methyladenosine (m^6^A) is involved in the development of drug resistance in various cancer types. The role of N6-methyladenosine (m^6^A) methyltransferase, KIAA1429, in the resistance of gastric cancer to cisplatin is largely unknown. In this study, the KIAA1429 expression level as well as m^6^A content were found to be higher in cisplatin resistant gastric cancer cells, and KIAA1429 regulated the sensitivity of gastric cancer cells to cisplatin treatment. We then identified p65 as the regulator of KIAA1429 expression. Mechanistically, KIAA1429 regulated the sensitivity of gastric cancer cells to cisplatin by stabilizing FOXM1 mRNA via YTHDF1. The findings from this study suggest that KIAA1429 could be a therapeutic target of cisplatin resistance in gastric cancer.

**Abstract:**

Although cisplatin is frequently used to treat gastric cancer, the resistance is the main obstacle for effective treatment. mRNA modification, N6-methyladenosine (m^6^A), is involved in the tumorigenesis of many types of cancer. As one of the largest m^6^A methyltransferase complex components, KIAA1429 bridges the catalytic m^6^A methyltransferase components, such as METTL3. In gastric cancer, KIAA1429 was reported to promote cell proliferation. However, whether KIAA1429 is involved in the resistance of gastric cancer to cisplatin remains unclear. Here, we generated cisplatin resistant gastric cancer cell lines, and compared the m^6^A content between resistant cells and wild type cells. The m^6^A content as well as KIAA1429 expression are higher in resistant cells. Interestingly, the expression of KIAA1429 was significantly increased after cisplatin treatment. We then used shRNA to knockdown KIAA1429 and found that resistant cells responded more to cisplatin treatment after KIAA1429 depletion, while overexpression of KIAA1429 decreased the sensitivity. Moreover, we identified a putative p65 binding site on the promoter area of KIAA1429 and ChIP assay confirmed the binding. p65 depletion decreased the expression of KIAA1429. YTHDF1 is the most abundant m^6^A “reader” that interacts with m^6^A modified mRNA. Mechanistically, YTHDF1 was recruited to the 3′-untranslated Region (3′-UTR) of transcriptional factor, FOXM1 by KIAA1429 and stabilized FOXM1 mRNA. More importantly, KIAA1429 knockdown increased the sensitivity of resistant cells to cisplatin in vivo. In conclusion, our results demonstrated that KIAA1429 facilitated cisplatin resistance by stabilizing FOXM1 mRNA in gastric cancer cells.

## 1. Introduction

Although the incidence of gastric cancer (GC) is decreasing, it remains the second leading cause of cancer-related death in China [1]. Most GC patients have no early symptoms, which causes a high percentage of patients to be at an advanced stage with lymph nodes or distant organ metastases when diagnosed. Chemotherapy remains the first-line regime for advanced GC. Among the chemotherapeutic drugs, cisplatin is widely used because of its effectivity for treating GC [2]. However, the effectivity of cisplatin is usually impaired by intrinsic or acquired resistance. Thus, understanding the mechanism of resistance and discovering new therapeutic targets that could improve cisplatin sensitivity in GC is essential.

N^6^ -methyladenosine (m^6^A) is the most abundant RNA modification that is regulated by “writers” (methyltransferases), “erasers” (demethylases), and “readers” (effector proteins) [3,4]. In eukaryotes, m^6^A modification exerts multiple functions including mRNA stability, exporting, splicing, and translation [5,6,7,8,9,10]. The methyltransferase is a complex that consists of methyltransferase-like 3 (METTL3), WT1-associated protein (WTAP), vir-Like m^6^A methyltransferase-associated (KIAA1429), and methyltransferase-like 14 (METTL14) [11]. Increasing evidence has demonstrated that m^6^A machinery plays a crucial role in cancer development. For instance, METTL14 promotes pancreatic cancer cell proliferation and migration, which relies on its methyltransferase activity [12]. Recently, accumulating evidence has indicated that METTL3 is involved in regulating cell growth and migration in multiple tumor types, such as liver cancer, GC, acute myeloid leukemia (AML), pancreatic cancer, breast cancer, and lung cancer, in an either dependent or independent m^6^A methyltransferase activity manner [13]. KIAA1429 is the largest component and biochemical study revealed that KIAA1429 could recruit other three catalytic components, METTL14/WTAP/METTL3, to the RNA substrates to perform m^6^A modification [14]. In methyltransferase complex, KIAA1429 bridges catalytic core components METTL3 and METTL14. Interestingly, KIAA1429 depletion decreased about ∼4-fold m^6^A content and this is significantly more prominent than METTL3 or METTL14 knockdown, which suggests that KIAA1429 plays important role in methyltransferase complex and could be involved in tumorigenesis [15]. KIAA1429 has been shown to facilitate the advancement and metastasis of hepatocellular carcinoma (HCC) in an m^6^A-dependent manner [16]. In breast cancer, KIAA1429 has been found to act as an oncogenic factor and promote cells growth in an m^6^A-independent manner [17]. In GC, KIAA1429 stabilizes c-Jun mRNA to promote cancer cell proliferation [18], and stabilizes GLUT1 mRNA transcript stability to accelerate GC aerobic glycolysis in an m^6^A-dependent manner [19]. Although being reported closely related to cancer progression, the importance of KIAA1429 in regulating drug resistance in GC needs to be investigated.

m^6^A methyltransferases have been reported to stabilize the mRNA of forkhead transcription factor family members, including forkhead box M1 (FOXM1) in various diseases [20,21]. FOXM1 is highly expressed in several cancer types, such as GC, colorectal cancer, liver cancer, lung cancer, prostate cancer, and breast cancer [22,23,24,25,26,27]. Patients with overexpressed FOXM1 show less sensitivity to chemotherapy, such as platinum drugs [28,29]. Furthermore, the expression of FOXM1 has been negatively correlated with patient prognosis in gastric cancer [30]. In this study, we demonstrate that KIAA1429 is overexpressed in cisplatin resistant GC cells and KIAA1429 regulated the sensitivity of GC cells to cisplatin by stabilizing the FOXM1 mRNA, which provides evidence for a potential therapeutic regimen to overcome drug resistance.

## 2. Materials and Methods

### 2.1. Antibodies

Anti-p65 (#8242, 1:1000), p-p65 (#3033, 1:500), METTL14 (#48699, 1:1000), WTAP (#41934, 1:1000), METTL3 (#86132, 1:1000), and GAPDH (#5174, 1:3000) antibodies were purchased from Cell Signaling (MA, USA); anti-KIAA1429 (#PA5-95717, 1:1000), YTHDF1 (#17479-1-AP, 1:500), and m^6^A (#MA5-33030, 1:200) antibodies were obtained from ThermoFisher Scientific (Waltham, MA, The United States of America (USA)).

### 2.2. Plasmid Overexpression Experiment

p65, KIAA1429, and YTHDF1 plasmids were used for overexpression. Plasmid for p65 overexpression was obtained from addgene [31]; pCMV6-Entry, KIAA1429, and YTHDF1 overexpression plasmids were purchased from Origene (Rockville, MD, USA). Briefly, 0.5 × 10^6^ gastric cancer cells were seeded into a 6-well plate. Empty vector(pCMV6-Entry), p65, KIAA1429, or YTHDF1 plasmids were diluted in Opti-MEM and mixed with Lipofectamine 2000 transfection reagent (Invitrogen, Waltham, MA, USA). After incubation for 5 min at room temperature, the mixture was added into a 6-well plate and the cells were incubated for 24 h at 37 °C.

### 2.3. Tissue Culture

The human gastric cancer cell lines, NCI-N87 (RRIDs: CVCL_1603) and AGS (RRIDs: CVCL_0139), were purchased from ATCC (American Type Culture Collection, USA). The cells were cultured in DMEM/F12 (Invitrogen, CA, USA) medium that supplemented with 10% (*v/v*) of heat inactivated FBS (Gibco, USA) at 37 °C with 5% CO_2_. To generate cisplatin resistant GC cell lines, NCI-N87 and AGS cells were treated with gradually increasing concentrations of cisplatin (Sigma-Aldrich, MS, USA) for about two months until the cells could grow in the medium supplemented with 1000 nM cisplatin. The resistant cell lines were named AGS Cis-R and NCI-N87 Cis-R.

### 2.4. Short Hairpin RNA (shRNA) Knockdown

The control plasmid pLKO.1-puro, shRNA vectors for KIAA1429, YTHDF1, and p65 were purchased from Sigma-Aldrich (Burlington, MA, USA). To produce the lentiviral particles, shRNA plasmids were co-transfected with packaging constructs (pMD2.G and psPAX2, Addgene) in HEK293T cells, and the medium was harvested. To knockdown KIAA1429, p65, or YTHDF1, GC cells were infected with lentiviral particles in the presence of 8 μg/mL polybrene (Sigma-Aldrich, USA), the medium was changed after 24 h incubation. The cells were then incubated in the fresh medium for another 48 h.

### 2.5. Cell Viability

CCK8 kits (Dojindo, Kumamoto, Kyushu, Japan) were used for cell viability analysis. Briefly, 5000 GC cells were plated into a well of 96-well plate and treated with different concentrations of cisplatin for 72 h. A total of 10 μL CCK8 solution was added into each well and the plate was incubated in the incubator for 4 h. The results were obtained by measuring the absorbance at 450 nm using SpectraMax (Molecular Devices, San Jose, CA, USA).

### 2.6. m^6^A RNA Methylation Quantification

An m^6^A RNA Methylation Quantification Kit (Abcam, Cambridge, MA, USA) was used to determine m^6^A content in total RNA. Briefly, 2 μL of negative control, 2 μL of diluted positive control RNA, and a 200 ng sample RNA were added into each well that contained RNA binding solution. After incubation at 37 °C for 90 min, the wells were washed with wash buffer three times. Diluted capture antibody was then added to capture m^6^A, followed by the addition of diluted detection antibody. An m^6^A signal was obtained by adding enhancer solution and developer solution and reading the absorbance at a wavelength of 450 nm using SpectraMax (Molecular Devices, San Jose, CA, USA).

### 2.7. Quantitative Reverse Transcription-PCR (QRT-PCR) Experiment

The total RNA was extracted using an RNeasy Plus Mini Kit (Qiagen, Germantown, MD, USA) according to the standard procedures. The first-strand cDNA was prepared using a QuantiTect reverse transcription kit (Qiagen, Germantown, MD, USA) according to manufacturer’s instructions. Quantitative reverse transcription-PCR was performed to measure KIAA1429 and FOXM1 mRNA level using LightCycler^®^ 480 SYBR Green I Master (Roche Life Science, USA) with the following primers: KIAA1429, forward: 5′-TGACCTTGCCTCACCAACTGCA-3′, reverse: 5′-AGCAACCTGGTGGTTTGGCTAG-3′; FOXM1, forward: 5′-TCTGCCAATGGCAAGGTCTCCT-3′, reverse: 5′-CTGGATTCGGTCGTTTCTGCTG-3′; The reaction was carried out with the following conditions: 94 °C for 10 s, 60 °C for 30 s, 68 °C for 2 min, 38 cycles. The melting curve was used to confirm single amplicons. GAPDH, forward: 5′- AATCCCATCACCATCTTCCAG-3′, reverse: 5′-AAATGAGCCCCAGCCTTC-3′. GAPDH was used as an endogenous control. The relative expressions were calculated by applying the 2−^ΔΔ^^Ct^ approach [32].

### 2.8. mRNA Stability Assay

Briefly, 3 × 10^5^ AGS Cis-R and NCI-N87 Cis-R cells were seeded in each well of a 6-well plate before being infected with scramble, KIAA1429 or YTHDF1 shRNA. After being treated with 5 μg/mL Actinomycin D (Sigma-Aldrich, USA) for different time points, the cells were harvested. The total RNA was extracted using a RNeasy Plus Mini Kit and 1 μg total RNA was reversed to cDNA. FOXM1 mRNA level was then determined by QRT-PCR assay.

### 2.9. Western Blotting

The RIPA buffer (Invitrogen, Waltham, MA, USA) that was supplemented with protease and phosphatase inhibitors (Roche, South San Francisco, CA, USA) was used to break GC cells. After quantification with a BCA protein assay kit (Pierce, Waltham, MA, USA), samples of protein (20 μg) were separated by SDS-PAGE gel, and transferred to nitrocellulose membrane (Invitrogen, Waltham, MA, USA), which was blocked in 5% non-fat milk (Bio-Rad, Hercules, CA, USA) at room temperature for 1 h, reacted with the primary antibodies in 5% non-fat milk at 4 °C overnight, and incubated with secondary antibodies (in 5% non-fat milk) at room temperature for 1 h. The blots were developed using Super Signal Chemiluminescent Substrate (Thermo Scientific, Waltham, MA, USA) and were visualized with Las4000 system (GE Healthcare, Chicago, IL, USA). The densitometric quantification was performed using ImageJ software.

### 2.10. Chromatin Immunoprecipitation (ChIP) Assay

ChIP assays were used to investigate the binding of p65 on KIAA1429 promoter. To perform ChIP assays, SimpleChIP^®^ Enzymatic Chromatin IP Kit (Cell Signaling Technologies, USA) was used. Briefly, 1 × 10^7^ wild type or cisplatin resistant GC cells were incubated with 1% formaldehyde to crosslink proteins to DNA and the reaction was stopped by adding glycine solution. Micrococcal nuclease was used to digest the chromatin into 150–900 bp length range. A total of 4 µg anti-p65 antibody was mixed with prepared chromatin and incubated at 4 °C with rotation overnight. After incubation with protein G magnetic beads at 4°C for 2 h, the tube was placed at 65 °C to elute protein-DNA complex from the beads. Proteinase K was then added to reverse the cross-links. The pulled down DNA was subjected to QRT-PCR experiments with the specific primers for KIAA1429 promoter: forward: 5′-TACGTGGGCGAGAATTTTCCT-3′, reverse: 5′-TGAGCTTTGGAGCAACGAGA-3.

### 2.11. RNA-Immunoprecipitation (RIP) and MeRIP

RIP was used to investigate the protein–RNA interaction (YTHDF1 and FOXM1 mRNA) and MeRIP was used to determine the m^6^A modification on FOXM1 mRNA. To determine the m^6^A modification on mRNA and protein–RNA interaction, RIP and MeRIP assays were performed using the Magna RNA-binding Protein Immunoprecipitation Kit (Millipore, Burlington, MA, USA). RIP lysis buffer was used to lyse GC cells. Before the addition of protein G dynabeads to the lysate, 5 μg m^6^A or YTHDF1 antibodies were added and incubated at 4 °C with rotation overnight. Proteinase K was used to elute the RNA, which was then purified using the RNeasy Mini Kit. The pulled down RNA was subjected to QRT-PCR experiments with the specific primers: FOXM1 motif, forward: 5′-CCTCTGAGTGAGGACAGCAG-3′, reverse: 5′-AACACAAGGTCCCAGCAGTG-3.

### 2.12. Mouse Studies

All mice experiments were performed following the NIH Guide for the Care and Use of Laboratory Animals [33]. The six-week-old NOD/SCID mice were purchased from The Jackson Laboratory, mice were divided into four groups (n = 6 per group). After infected with KIAA1429 or scramble shRNA, 5.0 × 10^6^ AGS Cis-R or NCI-N87 Cis-R cells were subcutaneously injected into the flank of the mice. The mice were treated with the same volume of vehicle or 5 mg/kg cisplatin intraperitoneally three times a week after palpable tumors were observed. Tumor growth was monitored by measuring the tumor size using caliper and calculated using the formula: V = ½ (Length × Width^2^) every three days. The mice were euthanized once the tumor size reached the end point (2000 mm^3^). 

### 2.13. Statistical Analysis

Data are means ± SD (standard deviations) from three independent experiments. R statistical software (version 4.1.1) was used for statistical analyses. The Student’s *t*-test was used for the comparison of two groups; one-way analysis of variance (ANOVA) was used for comparison of multiple groups, and the two-way ANOVA was used to compare tumor growth. The significance is defined as *p* value ˂ 0.05.

## 3. Results

### 3.1. KIAA1429 Was Highly Expressed in Cisplatin Resistant GC Cells

**Figure 1:**
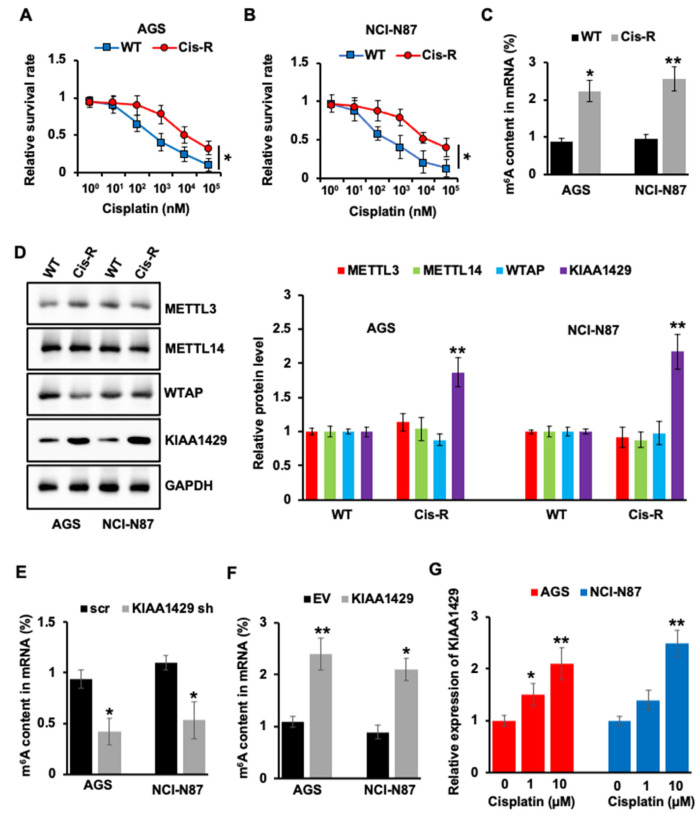
Effect of cisplatin on KIAA1429 expression. (**A**,**B**) AGS Cis-R (A)/NCI-N87 Cis-R (**B**) and their parental wild type (WT) cells were treated with indicated concentration of cisplatin for 72 h, cell survival was assessed by CCK-8 assay. (**C**) m^6^A quantitative analysis showed the percentage of m^6^A content in AGS Cis-R or NCI-N87 Cis-R and their parental wild type cells. (**D**) The protein level of METTL3, METTL14, WTAP, and KIAA1429 in WT and Cis-R cells were analyzed by Western blotting and densitometric quantification. (**E**,**F**) m^6^A quantitative analysis showed the percentage of m^6^A content in KIAA1429 shRNA infected (**E**) or KIAA1429 overexpressed cells (**F**). (**G**) Gastric cancer cells were treated with different doses of cisplatin for 48 h, the mRNA level of ki-aa1429 was assessed by QRT-PCR. * *p* < 0.05, ** *p* < 0.01.

Cisplatin remains major chemotherapeutic drug in gastric cancer. However, primary or acquired resistance decreases the affectivity of cisplatin. To explore the mechanism of cisplatin resistance, resistant GC cell lines, AGS Cis-R, and NCI-N87 Cis-R were generated. Cell survival experiments showed that AGS Cis-R and NCI-N87 Cis-R presented a significantly (*p* = 0.0173 and *p* = 0.0207) reduced response to cisplatin compared to wild type (WT) cells (Figure 1A,B). m^6^A modification was proven to be closely related to drug resistance [34,35]; we then compared m^6^A content between resistant and wild type GC cells to study the involvement of m^6^A modification in drug resistance. The m^6^A content in mRNA is significantly (*p* = 0.0112 and *p* = 0.0018) higher in cisplatin resistant GC cells (Figure 1C). Next, we wanted to compare the expression level of m^6^A methyltransferases in cisplatin resistant and wild type GC cells. Interestingly, only KIAA1429 showed higher expression in cisplatin resistant cells, but not METTL3, METTL14, and WTAP (Figure 1D). Furthermore, KIAA1429 depletion decreased m^6^A content in mRNA (Figure 1E), and overexpression of KIAA1429 increased m^6^A content (Figure 1F), which suggested that cisplatin regulated m^6^A content through KIAA1429. This hypothesis was further confirmed by the experiment, which showed that cisplatin treatment increased KIAA1429 expression (Figure 1G). 

### 3.2. KIAA1429 Regulated the Sensitivity of GC Cells to Cisplatin

**Figure 2:**
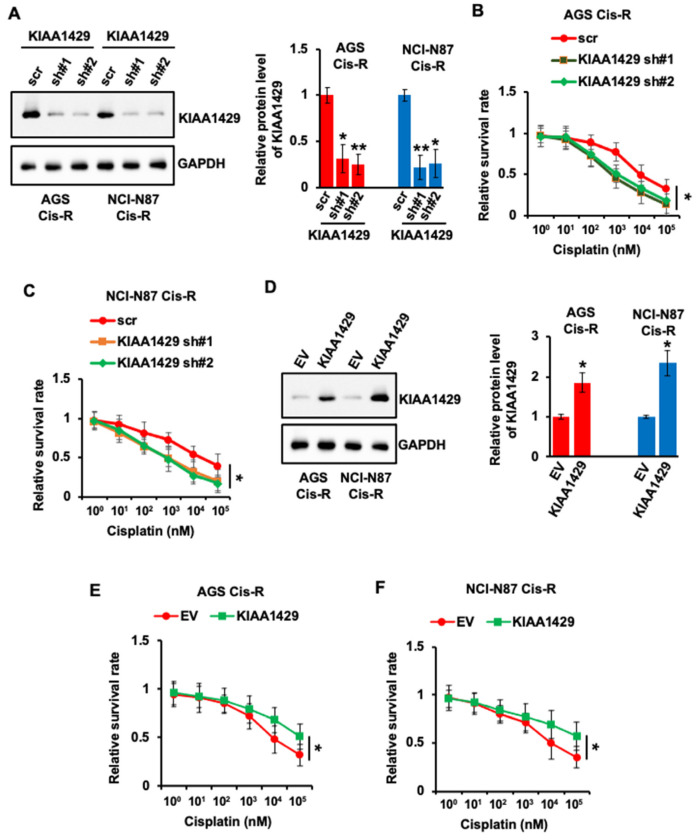
Effect of KIAA1429 depletion or overexpression on cisplatin resistance in gastric cancer cells. (**A**) AGS Cis-R or NCI-N87 Cis-R cells were infected with KIAA1429 shRNA, and the protein level of KIAA1429 was assessed by Western blotting and densitometric quantification. (**B**,**C**) Cisplatin IC50 experiment was performed in AGS Cis-R (**B**) or NCI-N87 Cis-R (**C**) cells after infected with KIAA1429 shRNA. (**D**) AGS Cis-R or NCI-N87 Cis-R cells were transfected with KIAA1429 plasmid, and the protein level of KIAA1429 was assessed by Western blotting and densitometric quantification. (**E**,**F**) Cisplatin IC50 experiment was performed in AGS Cis-R (**E**) or NCI-N87 Cis-R (FC) cells after transfected with KIAA1429 plasmid. * *p* < 0.05, ** *p* < 0.01.

Cisplatin treatment increased the expression of KIAA1429; we wondered whether KIAA1429 was involved in chemoresistance. First, we performed knockdown of KIAA1429 using two specific shRNAs in cisplatin resistant GC cells and about 80% KIAA1429 was depleted in both cell lines (Figure 2A). Importantly, the sensitivity of both resistant cell lines to cisplatin increased after KIAA1429 depletion (Figure 2B,C).

We then wondered whether KIAA1429 overexpression decreased the sensitivity. To achieve that, AGS Cis-R and NCI-N87 Cis-R cells were transfected with KIAA1429 plasmid or empty vector. The protein level of KIAA1429 increased more than two times in the cells transfected with KIAA1429 plasmid compared with cells transfected with empty vector (Figure 2D). Not surprisingly, KIAA1429 overexpression decreased the response of resistant cells to cisplatin (Figure 2E,F).

In addition, we tested the effect of KIAA1429 depletion or overexpression on the sensitivity of wild type GC cells to cisplatin treatment. As shown in Figure S1A–C, KIAA1429 depletion increased the response of wild type AGS and NCI-N87 cells to cisplatin, while KIAA1429 overexpression decreased the sensitivity of wild type GC cells to cisplatin (Figure S1D–E).

### 3.3. Expression of KIAA1429 Was Regulated by Transcriptional Factor p65 in GC Cells

**Figure 3:**
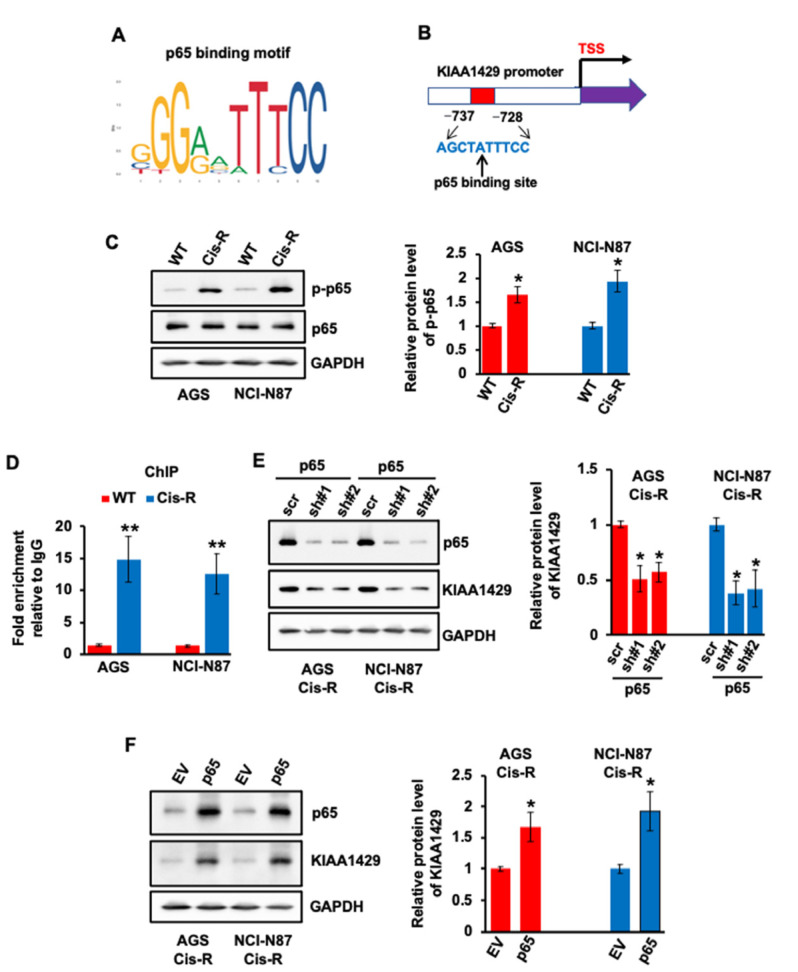
Effect of p65 on regulating KIAA1429 expression. (**A**) The canonical binding motif of p65. (**B**) Schematic diagram of KIAA1429 promoter region con-taining one putative p65 binding sites. (**C**) The phosphorylation of p65 and total p65 levels in AGS Cis-R or NCI-N87 Cis-R and their parental wild type cells was assessed by Western blotting and densitometric quantification. (**D**) ChIP assay indicated an increase of p65 binding to KIAA1429 promoter in AGS Cis-R or NCI-N87 Cis-R compared with their parental wild type cells. (**E**) The protein level of KIAA1429 was assessed by Western blotting and densitometric quantification after p65 depletion. (**F**) The protein level of KIAA1429 was assessed by Western blotting and densitometric quantification after p65 overexpression. * *p* < 0.05, ** *p* < 0.01.

The mechanism of how KIAA1429 was up-regulated in cisplatin resistant GC cells remains unknown. p65, an important transcriptional factor, has been proven to be involved in cisplatin resistance in multiple cancer types, including GC [36,37]. In addition, p65 bound the promoter area of METTL14, and regulated its expression in pancreatic cancer cells [38]. Thus, we hypothesized that KIAA1429 could also be regulated by p65. One putative p65 binding motif was found after analyzing the promoter sequence of KIAA1429 (Figure 3A,B). The activity of p65 relies on its phosphorylation; we then compared the phosphorylation level of p65 in WT and cisplatin resistant cells. As shown in Figure 3C, the phosphorylation level of p65 was obviously higher in resistant GC cell lines compared with WT cells. No obvious change was observed with total p65. These data suggested that higher activity of p65 could be the cause of KIAA1429 up-regulation in cisplatin resistant cells. To prove this, p65 antibody was used for ChIP assay. More than 10 times the p65 binding (−737~−728) was observed in resistant GC cells compared with WT cells (Figure 3D). More importantly, p65 depletion decreased the protein level of KIAA1429 (Figure 3E), which was then increased after p65 overexpression (Figure 3F). These data indicated that p65 regulates the expression of KIAA1429 via binding to its promoter in GC cells. 

### 3.4. KIAA1429 Maintained the FOXM1 mRNA Stability in Gastric Cancer Cells

**Figure 4:**
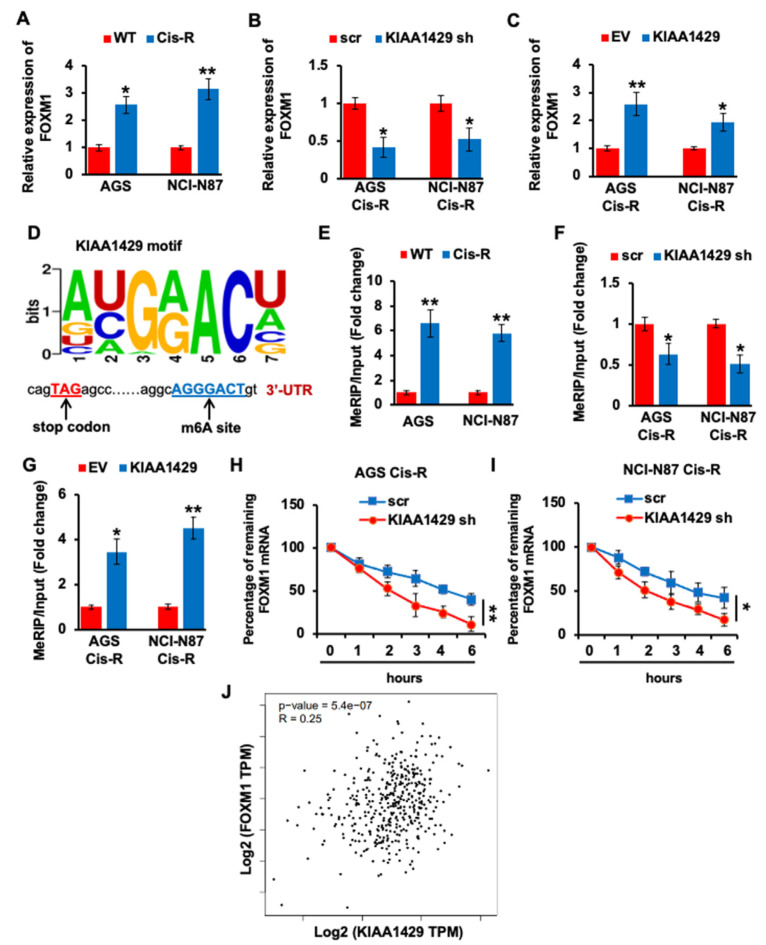
Effect of KIAA1429 on regulating FOXM1 expression. (**A**) The mRNA level of FOXM1 in AGS Cis-R or NCI-N87 Cis-R and their parental wild type cells was assessed by QRT-PCR. (**B**,**C**) The mRNA level of FOXM1 in KIAA1429 shRNA infected (**B**) or KIAA1429 overexpressed (**C**) AGS Cis-R or NCI-N87 Cis-R cells was assessed by QRT-PCR. (**D**) Schematic diagram demonstrated the m^6^A motif of KIAA1429 and the m^6^A site in the 3′-UTR of FOXM1 mRNA (near stop codon). (**E**) MeRIP assay indicated the FOXM1 mRNA enrichment precipitated by m^6^A antibody in AGS Cis-R or NCI-N87 Cis-R and their parental wild type cells. (**F**,**G**) MeRIP assay indicated the FOXM1 mRNA enrichment precipitated by m^6^A antibody in KI-AA1429 shRNA infected (**F**) or KIAA1429 overexpressed (G) AGS Cis-R or NCI-N87 Cis-R cells. (**H**,**I**) RNA decay rate assay demonstrated the FOXM1 mRNA half-lives upon the KIAA1429 knockdown in AGS Cis-R (**H**) or NCI-N87 Cis-R (**I**) cells. Data were detected at indicated timepoint with actinomycin D (Act D, 5 μg/mL) treatment. (**J**) Correlation analysis by Spearman’s rank correlation coefficient (GEPIA) showed the correlation between KIAA1429 and FOXM1 in the gastric cancer tissue specimens. * *p* < 0.05, ** *p* < 0.01.

As a transcriptional factor, FOXM1 plays multiple roles in tumorigenesis and development, such as promoting cell differentiation and proliferation, facilitating tumor metastasis, and invasion [39]. In addition, higher FOXM1 expression predicted less response of GC patients to cisplatin [40]. Consistent with other studies, the mRNA level of FOXM1 is significantly (*p* = 0.0105 and *p* = 0.0013) higher in cisplatin resistant GC cells (Figure 4A). To investigate whether the expression of FOXM1 could be regulated by KIAA1429, we performed knockdown on KIAA1429 using shRNA and found that KIAA1429 depletion significantly (*p* = 0.0109 and *p* = 0.0139) decreased FOXM1 mRNA level, and KIAA1429 overexpression increased FOXM1 mRNA level (Figure 4B,C). Since KIAA1429 is one of the m^6^A methyltransferases, we analyzed the FOXM1 coding sequence (CDS) and identified an m^6^A motif of KIAA1429 in 3′-UTR (Figure 4D). We then performed MeRIP QRT-PCR and found that the m^6^A antibody pulled down more FOXM1 mRNA in cisplatin resistant GC cells (Figure 4E). In addition, KIAA1429 depletion decreased the FOXM1 mRNA pulled down by m^6^A antibody, and KIAA1429 overexpression increased the precipitated FOXM1 mRNA (Figure 4F,G). To investigate the effect of KIAA1429 on FOXM1 transcript stability, we performed RNA decay rate analysis and found that FOXM1 transcript half-life was decreased after KIAA1429 depletion (Figure 4H,I). Spearman rank correlation coefficient (GEPIA, http://gepia.cancer-pku.cn/, (accessed on 1 May 2022)) [41] was used to analyze the expression correlation between KIAA1429 and FOXM1. Although the scatter plot shows some linear trend, the correlation coefficient of 0.25 (less than 0.3) indicated negligible correlation between KIAA1429 and FOXM1 in GC samples (Figure 4J). All of the above data indicated that KIAA1429 regulated the expression of FOXM1 through m^6^A modification to stabilize the mRNA of FOXM1. 

### 3.5. YTHDF1 Regulated the FOXM1 mRNA Stability in Gastric Cancer Cells

**Figure 5:**
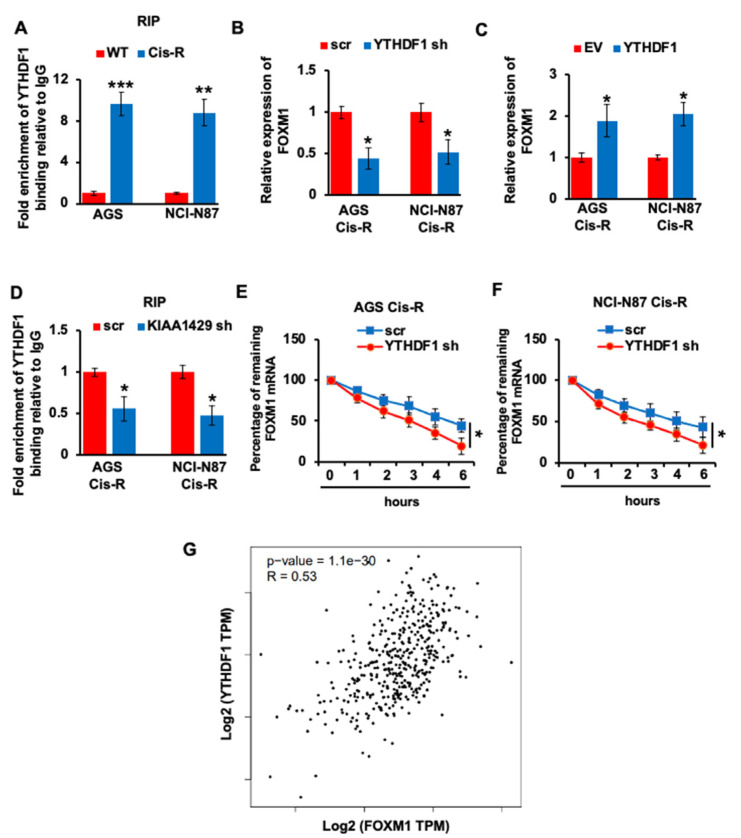
Effect of YTHDF1 on regulating FOXM1 expression. (**A**) RNA immunoprecipitation (RIP) indicated the direct binding within YTHDF1 and FOXM1 mRNA in AGS Cis-R or NCI-N87 Cis-R and their parental wild type cells. (**B**,**C**) The mRNA level of FOXM1 in YTHDF1 shRNA infected (**B**) or YTHDF1 overexpressed (**C**) AGS Cis-R or NCI-N87 Cis-R cells was assessed by QRT-PCR. (**D**) RNA immunoprecipitation (RIP) indicated the direct binding within YTHDF1 and FOXM1 mRNA in KIAA1429 shRNA infected AGS Cis-R or NCI-N87 Cis-R cells. (**E**,**F**) RNA decay rate assay demonstrated the FOXM1 mRNA half-lives upon the YTHDF1 knockdown in AGS Cis-R (**E**) or NCI-N87 Cis-R (**F**) cells. Data were detected at indicated timepoint with actinomycin D (Act D, 5 μg/mL) treatment. (**G**) Correlation analysis by Spearman’s rank correlation coefficient (GEPIA) showed the correlation between YTHDF1 and FOXM1 in the gastric cancer tissue specimens. * *p* < 0.05, ** *p* < 0.01, *** *p* < 0.001.

Although we proved that the expression of FOXM1 is regulated by KIAA1429, the underlying mechanism by which KIAA1429 stabilized the mRNA of FOXM1 remains unclear. YTH m^6^A RNA binding protein (YTHDF1) is one of the “readers” of m^6^A methyltransferase. We first performed RNA immunoprecipitation (RIP) assay and found that YTHDF1 bound the same m^6^A motif with KIAA1429 in FOXM1 CDS, and more binding was observed in cisplatin resistant GC cells (Figure 5A). More importantly, YTHDF1 depletion decreased the mRNA level of FOXM1, while YTHDF1 overexpression increased FOXM1 mRNA expression (Figure 5B,C). Furthermore, KIAA1429 knockdown significantly decreased the interaction between FOXM1 mRNA and YTHDF1, which suggested that the interaction between FOXM1 mRNA and YTHDF1 relies on the m^6^A modification generated by KIAA1429 (Figure 5D). To investigate the effect of YTHDF1 on FOXM1 transcript stability, we performed RNA decay rate analysis and found that the FOXM1 transcripts half-life was decreased after YTHDF1 depletion (Figure 5E,F). Furthermore, spearman rank correlation coefficient (GEPIA) was used to analyze the expression correlation between YTHDF1 and FOXM1, and the correlation coefficient of 0.53 indicated moderate positive correlation between YTHDF1 and FOXM1 in GC samples (Figure 5G). In conclusion, our results suggested that YTHDF1 recognized KIAA1429 methylated FOXM1 mRNA and KIAA1429/YTHDF1 enhanced the mRNA stability of FOXM1. 

### 3.6. KIAA1429 Depletion Increased Cisplatin Sensitivity In Vivo

**Figure 6:**
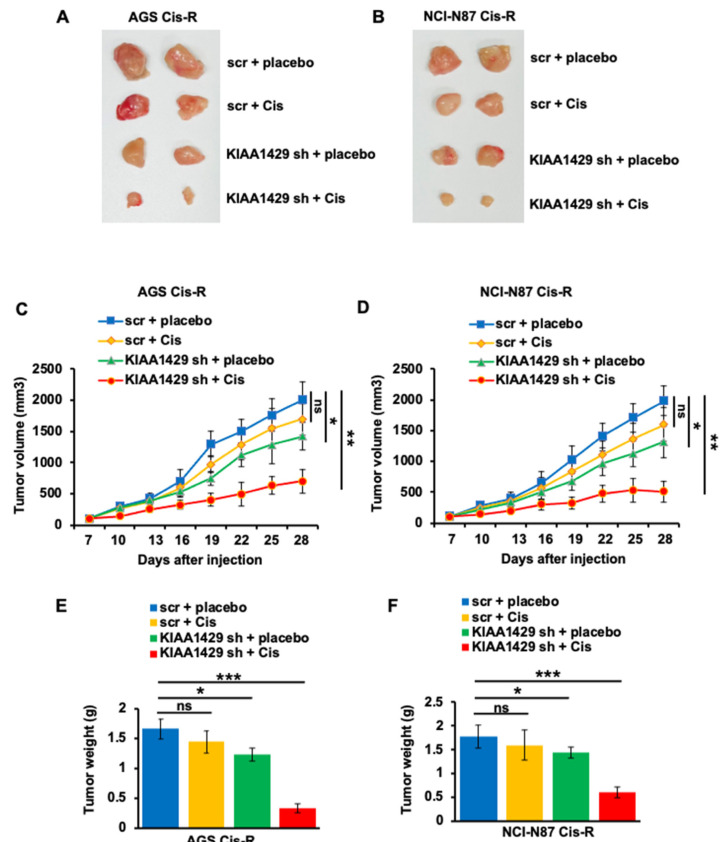
Effect of KIAA1429 depletion on cisplatin resistance in vivo. (**A**,**B**) Typical photos of tumors from each group. (**C**,**D**) Tumor growth curve indicated that KIAA1429 depletion significantly rescued the response of AGS Cis-R (**C**) and NCI-N87 Cis-R (**D**) cells to cisplatin. (**E**,**F**) The tumor weight of the subcutaneous grafts in each group at the end point after injection of AGS Cis-R (**E**) and NCI-N87 Cis-R (**F**) cells. * *p* < 0.05, ** *p* < 0.01, *** *p* < 0.001.

Our in vitro results indicated that KIAA1429 knockdown increased the sensitivity of GC cells to cisplatin. We then wonder whether KIAA1429 affected the sensitivity in a mouse model. To achieve this, we established a mouse model by subcutaneously injecting KIAA1429 or scramble shRNA infected AGS-Cis-R and NCI-N87 Cis-R cells into the flank of NOD/SCID mice. The mice were treated with the same volume of vehicle or 5 mg/kg cisplatin intraperitoneally three times a week after palpable tumors were presented. Consistent with our in vitro data, KIAA1429 depletion significantly blocked GC cell growth and the mice bearing scramble shRNA infected cells showed slight sensitivity to cisplatin. However, the mice bearing KIAA1429 shRNA infected cells showed higher sensitivity to cisplatin (Figure 6A–D). The tumors isolated from cisplatin-treated mice bearing cells that are infected with KIAA1429 shRNA are smaller than any of the other three groups (Figure 6E,F). 

## 4. Discussion

The main reason for GC treatment fails is the intrinsic or acquired resistance to the chemotherapy drugs. The affectivity of chemotherapy relies on the drugs itself and function-related genes [42], investigating the critical regulatory genes and their mechanism of action can provide the basis of a novel clinical therapeutic strategy for gastric cancer. As the most abundant RNA modification in eukaryotes, m^6^A modification exerts multiple functions including mRNA stability, export, splicing, and translation [5,6,7,8,9,10]. The m^6^A methyltransferase complex consists of METTL3, WTAP, METTL14, and KIAA1429. KIAA1429 is the largest component and biochemical study revealed that KIAA1429 could recruit the other three catalytic components, METTL14/WTAP/METTL3, to the RNA substrates to perform m^6^A modification [14]. Overexpression of methyltransferases components and m^6^A content has been identified in multiple cancer types, and this abnormal expression plays a critical role in chemotherapeutic drug resistance. In pancreatic cancer, METTL14 has been found to be involved in gemcitabine resistance [38]. METTL3 was also reported to regulate the sensitivity of melanoma cells to PLX4032 [43]. In non-small cell lung cancer (NSCLC), KIAA1429 was highly expressed and promoted NSCLC resistance to gefitinib [44]. To study the involvement of m^6^A methyltransferases in cisplatin resistance of GC, we compared the expression of METTL14, KIAA1429, METTL3, and WTAP in resistant and wild type GC cells. Surprisingly, only KIAA1429 was up-regulated in cisplatin resistant cells. More importantly, KIAA1429 depletion enhanced the response of GC cells to cisplatin, while KIAA1429 overexpression decreased the sensitivity.

Although KIAA1429 overexpression was found in multiple tumor types, little is known about the mechanism of how its expression is regulated. Transcriptional factor p65 was found to be involved in cisplatin resistance in several cancers. In addition, p65 regulated the gemcitabine resistance in pancreatic cancer via promoting the expression of METTL14 [36,37,38]. The activity of p65 relies on its phosphorylation, and p65 phosphorylation was found to be increased in our cisplatin resistant GC cells. By analyzing the promoter sequence of KIAA1429, we identified one p65 binding site. To prove that p65 could bind this motif, we performed ChIP experiments, and found that p65 bound KIAA1429 promoter in GC cells. Not surprisingly, the expression of KIAA1429 decreased after p65 knockdown by siRNA, and expression of KIAA1429 increased after p65 overexpression. These data indicated that the expression of KIAA1429 is regulated by p65 in GC cells.

As a member of the forkhead superfamily, FOXM1 regulates the tumorigenesis of various cancer types. The function of FOXM1 in determining sensitivity of cancer cells to chemotherapy drugs has also been reported [45]. In gastric cancer patients, overexpression of FOXM1 was related to cisplatin resistance, and depletion of FOXM1 improved this resistance [40,46]. FOXM1 was found to be up-regulated in cisplatin resistant GC cells, and KIAA1429 depletion significantly decreased FOXM1 expression. The consensus motif for the m^6^A site is *GGACU*, which is found in thousands of human transcripts. The major localization of m^6^A modification is revealed by transcriptome-wide mapping, which is near stop codon and in 3′-UTR [47]. Indeed, we analyzed FOXM1 CDS sequence and identified one m^6^A site with the sequence *GGACU,* near stop codon and in 3′-UTR. Therefore, we performed MeRIP assay to detect the m^6^A modification on FOXM1 mRNA. Higher m^6^A modification was found on FOXM1 mRNA in cisplatin resistant cancer cells. Importantly, KIAA1429 depletion decreased m^6^A modification and impaired the stability of FOXM1 mRNA, while KIAA1429 overexpressing increased m^6^A modification and FOXM1 mRNA stability. 

m^6^A performs functions by attracting “reader” proteins, and the YT521-B homology (YTH) family are the most widely studied proteins. There are five YTH family proteins, YTHDC1-2, and YTHDF1-3. YTH family proteins were reported to participate in RNA splicing via interacting with serine/arginine splicing factor, reducing the abundance of transcripts by promoting the translation of target RNAs, and regulating the stability of methylated mRNA transcripts [48,49,50]. YTHDF1 is the most abundant m^6^A “reader” that interacts with m^6^A modified mRNA to control the expression of certain genes. YTHDF1 was reported to be involved in regulating cancer cell growth, metastasis, and drug resistance via controlling the mRNA stability of cancer related genes. We found that YTHDF1 pulled down more FOXM1 mRNA in cisplatin resistant GC cells compared with wild type cells, perhaps because of more m^6^A modification on FOXM1 mRNA in resistant cells. YTHDF1 coordinates with other m^6^A regulators to promote cancer progression. In gastric cancer, YTHDF1 recognized m^6^A modified SPHK2 mRNA that was catalyzed by METTL3 [51]. Interestingly, the interaction between FOXM1 mRNA and YTHDF1 was significantly decreased after KIAA1429 depletion, which confirmed the collaboration between YTHDF1 and KIAA1429.

## 5. Conclusions

Collectively, we found that gastric cancer cells acquired cisplatin resistance by enhancing FOXM1 mRNA stability via KIAA1429 catalyzed m^6^A modification. Our findings suggest that KIAA1429 could be a new therapeutic target for improving the sensitivity of GC cells to cisplatin.

## Data Availability

Data supporting the reported results can be obtained from the corresponding author.

## Supplementary Materials

The following supporting information can be downloaded at: https://www.mdpi.com/article/10.3390/cancers14205025/s1, Figure S1: Effect of KIAA1429 depletion or overexpression on cisplatin response in wild type gastric cancer 2 cells. Figure S2: The Western blot figures in the Figure 1. Figure S3: The Western blot figures in the Figure 2. Figure S4: The Western blot figures in the Figure 3.
