# Supplementary material for: m6A Methyltransferase KIAA1429 Regulates the Cisplatin Sensitivity of Gastric Cancer Cells via Stabilizing FOXM1 mRNA
Source: Cancers (Basel). 2022 Oct 14;14(20):5025. doi: 10.3390/cancers14205025 (PMC9600291; doi:10.3390/cancers14205025)
Supplement: Supplementary file 1 [file cancers-14-05025-s001.zip › cancers-1916753-supplementary.pdf]

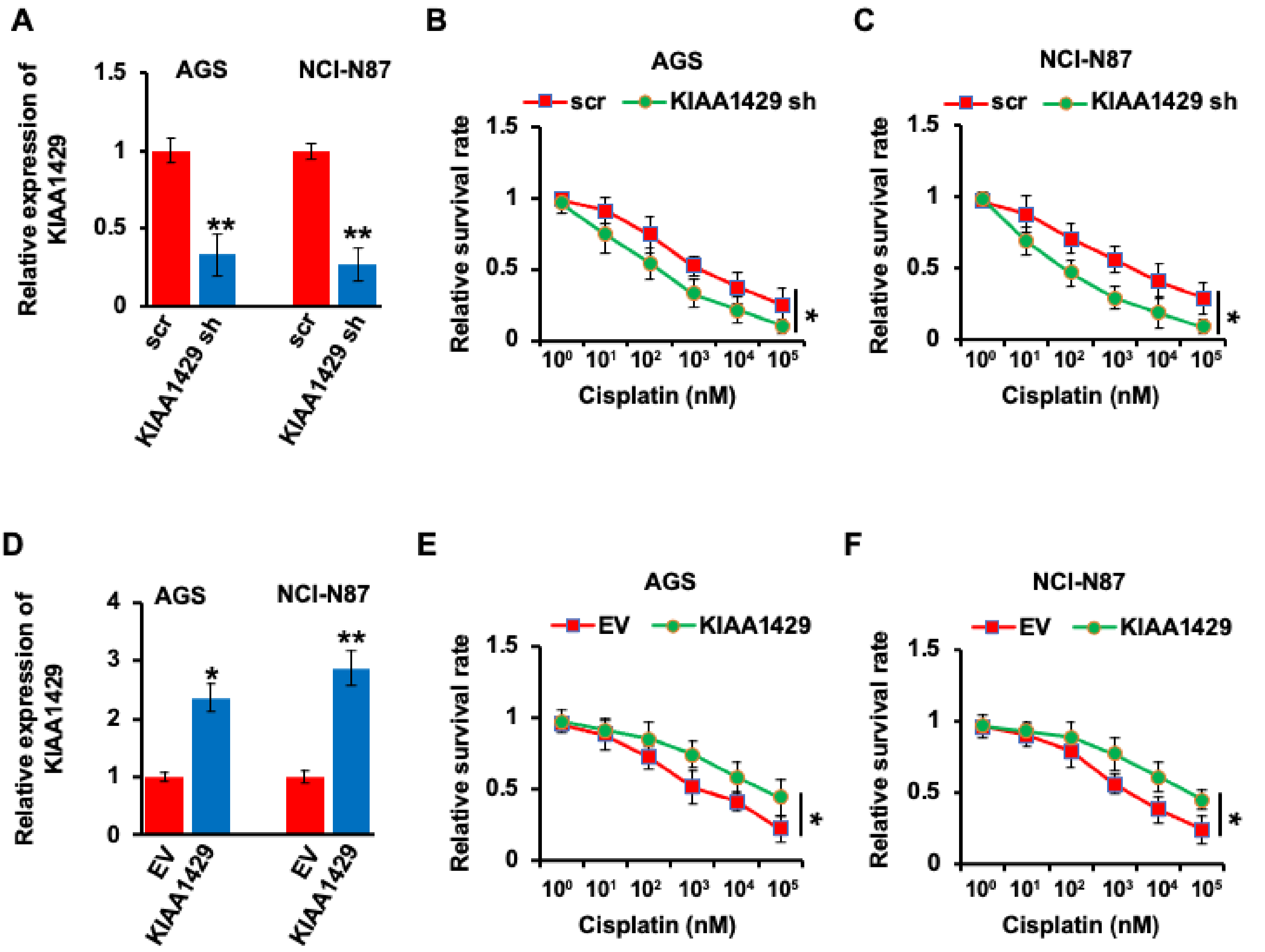

Figure S1. Effect of KIAA1429 depletion or overexpression on cisplatin response in wild type gastric cancer cells.

(A) AGS or NCI-N87 cells were infected with KIAA1429 shRNA, and the mRNA level of KIAA1429 was assessed by QRT-PCR. (B, C) Cisplatin IC50 experiment was performed in AGS (B) or NCI-N87 (C) cells after infected with KIAA1429 shRNA. (D) AGS or NCI-N87 cells were transfected with KIAA1429 plasmid, and the mRNA level of KIAA1429 was assessed by QRT-PCR. (E, F) Cisplatin IC50 experiment was performed in AGS (E) or NCI-N87 (F) cells after transfected with KIAA1429 plasmid. *p<0.05, **p<0.01.

Figure S2

Fig. 1D METTL3

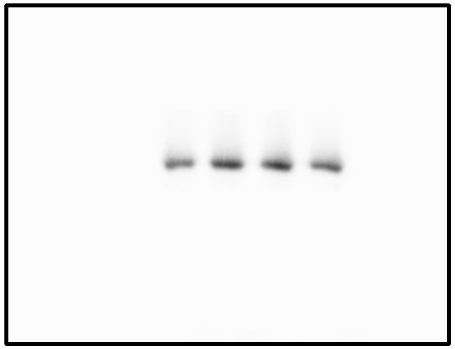

Fig. 1D METTL14

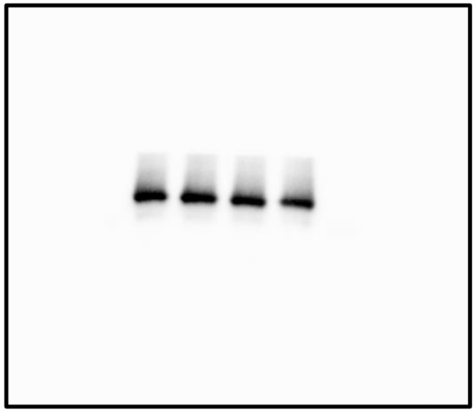

Fig. 1D WTAP

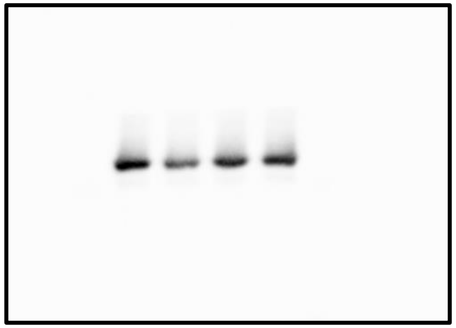

Fig. 1D KIAA1429

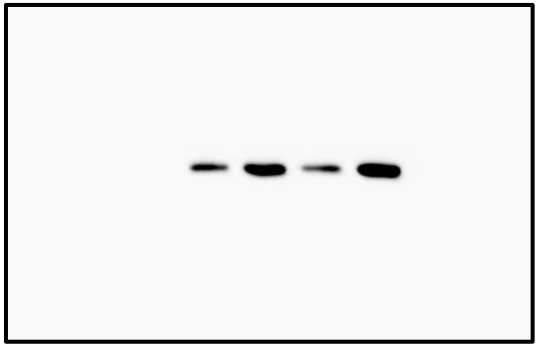

Fig. 1D GAPDH

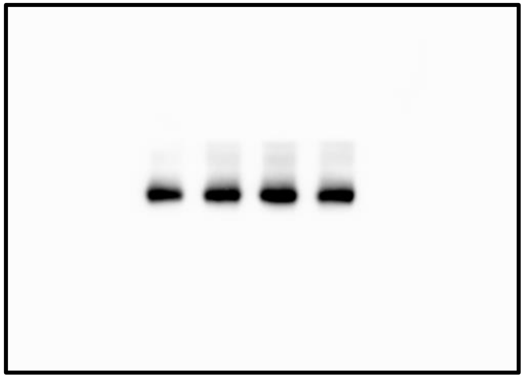

The Western Blot figures in the Figure 1.

Figure S3

Fig. 2A KIAA1429

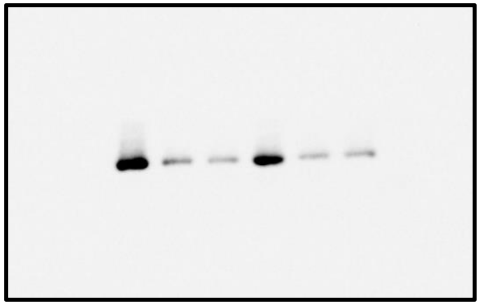

Fig. 2A GAPDH

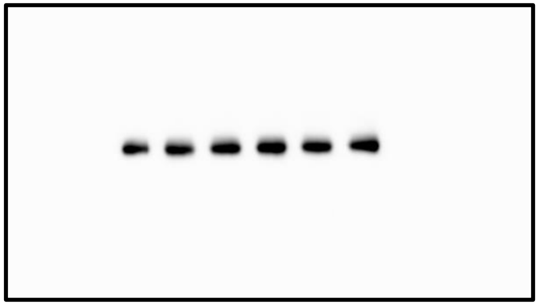

Fig. 2D KIAA1429

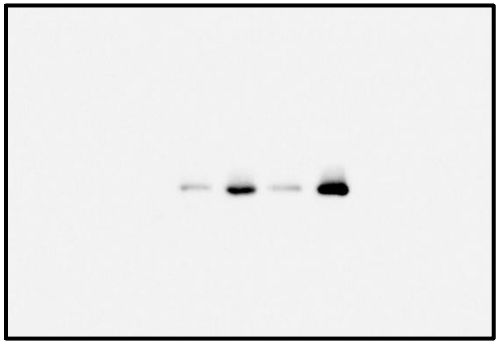

Fig. 2D GAPDH

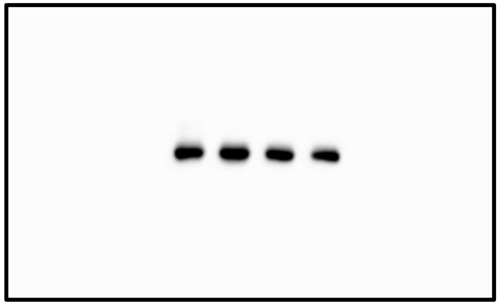

The Western Blot figures in the Figure 2.

Figure S4

Fig. 3C p-p65

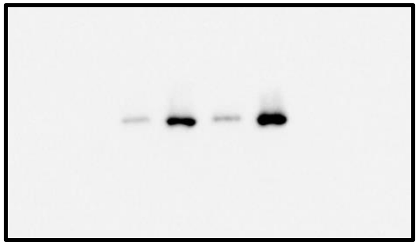

Fig. 3C p65

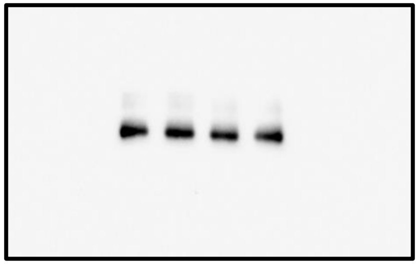

Fig. 3C GAPDH

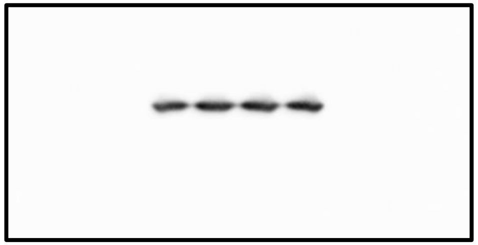

Fig. 3E p65

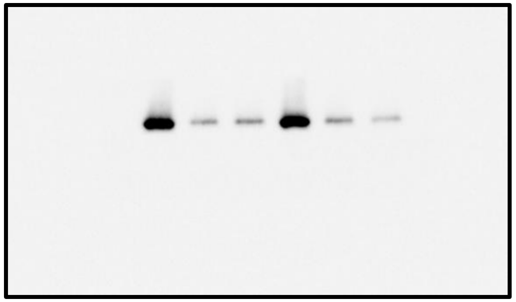

Fig. 3E KIAA1429

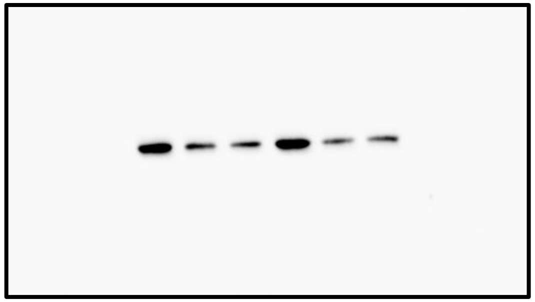

Fig. 3E GAPDH

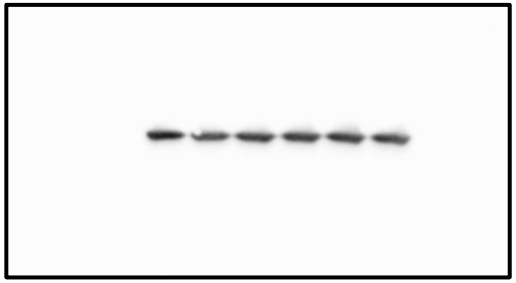

Fig. 3F p65

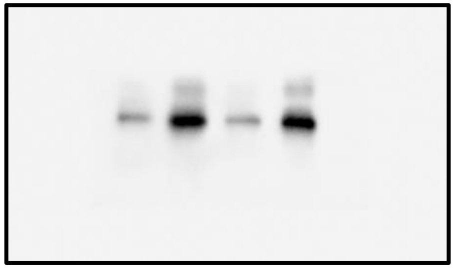

Fig. 3F KIAA1429

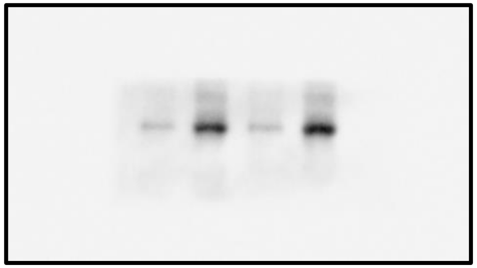

Fig. 3F GAPDH

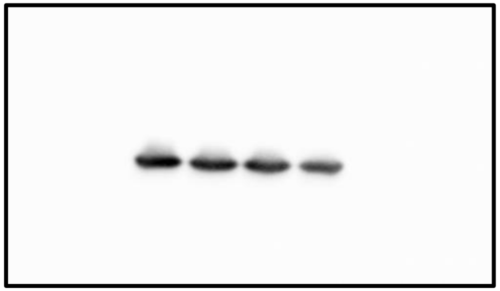

The Western Blot figures in the Figure 3.
